# One Species, Hundreds of Subspecies? New Insight into the Intraspecific Classification of the Old World Swallowtail (*Papilio machaon* Linnaeus, 1758) Based on Two Mitochondrial DNA Markers

**DOI:** 10.3390/insects13080752

**Published:** 2022-08-21

**Authors:** Paweł J. Domagała, Jerzy A. Lis

**Affiliations:** Institute of Biology, University of Opole, Oleska 22, 45-052 Opole, Poland

**Keywords:** Lepidoptera, Papilionoidea, *Papilio machaon*, mtDNA, haplotypes, intraspecific classification, subspecies, Palearctic Region

## Abstract

**Simple Summary:**

The Old World swallowtail *Papilio machaon* Linnaeus, 1758 is one of the most iconic butterflies, and like many butterflies, it was, and still is, a popular collecting item. *P. machaon* is characterised by a very high individual variability, which, combined with the great interest of collectors and amateur entomologists, has been converted into the descriptions of over 100 colour forms, aberrations and subspecies. The present study examines two mitochondrial DNA sequences (mtDNA) of 16S rDNA, and cytochrome b of 87 specimens of *P. machaon* collected in the entire Palearctic Region and their correlation with intraspecific classification. The results of our research showed that the proposed intraspecific classification by different authors does not correlate with the variability in mitochondrial DNA sequences.

**Abstract:**

The Old World swallowtail *Papilio machaon* Linnaeus, 1758 is one of the most well-known and most characteristic members of the family Papilionidae. Over the past two centuries, the butterfly has been the subject of many studies. *P. machaon* is characterised by a tendency to change the wing colour pattern. In turn, due to the great interest of collectors and amateur entomologists, these studies have been converted into the description of over 100 colour forms, aberrations and subspecies. In this study, mitochondrial DNA (mtDNA), 16S rDNA and cytochrome b sequences were used to examine the correlation between the intraspecific classification and genetic structure of *P. machaon*. The study used 87 specimens from 59 different localities covering the geographic distribution of this species in the Palaearctic. The phylogenetic relationships within and between the Old World swallowtail subspecies showed that the intraspecific classification proposed by various authors does not correlate with the variability in mitochondrial DNA sequences. In addition, populations occurring at the species distribution borders in the Palaearctic Region (i.e., Japan, Kamchatka, Morocco and Sakhalin) are genetically distinct from other species.

## 1. Introduction

The Old World swallowtail, *Papilio machaon* Linnaeus, 1758 is one of the most iconic butterflies. Over more than two centuries, the Old World swallowtail has been the subject of many studies, and its systematic position and biology have not raised any controversies. However, the intraspecific classification of this butterfly is still problematic.

*P. machaon* is a species with an extensive range of occurrences, including three zoogeographic regions and many habitats from subpolar and high mountains to semi-desert and subtropical [1,2,3]. Moreover, it is a species characterised by very high individual variability, which has resulted in descriptions of over 100 colour forms, aberrations and subspecies [1,2,3,4,5,6,7,8,9]. A list of colour forms, aberrations and subspecies of *P. machaon* is presented in Table S1. 

Over the past 200 years, many papers on the intraspecific classification of *P. machaon* have been published, but three of them are the most important. The first [1] provided a detailed analysis of *P. machaon* subspecies from the Old World. Its 35 races grouped into 13 groups were distinguished based on the analysis of the wing pattern and the copulatory structures [1]. A series of papers published by Seyer [2,7,8,9,10,11,12,13,14] is the second important study; the author attempted to explain the number of *P. machaon* subspecies and their relationship based on the wing patterns and the structure of the copulatory apparatus. Most importantly, these characteristics were analysed in individuals collected in nature and specimens obtained from crossbreeding individuals of different subspecies. As a result, the author distinguished 37 Old World *P*. *machaon* subspecies. Three illustrated colour atlases [3,15,16] are the most recent works. In these, hitherto recognised *P. machaon* subspecies are considered. Unfortunately, each presented subspecies is accompanied only by a very laconic morphological description in English [3] or Japanese [15,16]. 

In addition to the classic methods using morphological characteristics, molecular techniques have also been applied to resolve the question of intraspecific categories within *P. machaon*. The first studies focused on its North American populations and included allozyme polymorphism analysis [17] and restriction-fragment-length-polymorphism (RFLP) analysis [18,19]. 

However, the first two surveys using standard mitochondrial and nuclear DNA nucleotide sequences focused on the entire genus *Papilio* Linnaeus, 1758 [20,21], and only a few individuals of *P. machaon* were utilised. Most probably, a single specimen of this species, without specifying its place of origin, was included in the analyses of the 16S rDNA sequences and the sequences of the gene encoding ND1 [20]. On the other hand, *P. machaon* individuals from France and the United States were used to analyse the cytochrome oxidase I and II (COI and COII) sequences [21].

The first molecular approach focusing solely on *P. machaon* and other representatives of the *machaon* group was based on mitochondrial genes encoding COI/COII and the nuclear microsatellite sequences [22]. Although the analyses covered the North American representatives of the *machaon* group, several specimens of *P. machaon* subspecies from France (*P. m. gorganus*), the Czech Republic (*P. m. gorganus*) and Japan (*P. m. hippocrates*) were also included in the analyses.

In the subsequent molecular research [23,24,25], more specimens of *P. machaon* were included in analyses. Two of these studies [23,24] were based on the ND5 gene sequences. In both, examined specimens originated mainly from the Eastern Palearctic regions, including the Japanese Islands, where the genetic diversity of *P. machaon* was analysed [24]. Recently, during a study of European butterflies’ DNA barcodes [25], 168 specimens of this species were analysed. However, none of these analyses [23,24,25] considered the relation between DNA sequence variability and the intraspecific classification of *P. machaon*.

In the present study, we sequenced two mitochondrial DNA genes (16S rDNA and cytochrome b) of *P. machaon* specimens collected in various Palaearctic regions to determine the correlation of the sequence variability with the species intraspecific classification.

## 2. Materials and Methods

### 2.1. Subspecies Concept

The subspecies concept was first proposed by an ornithologist. In 1873, Elliott Coues [26] published “*A checklist of North American birds*”, in which he introduced a trinominal nomenclature, and a subspecies was abbreviated as var. 

In lepidopterology, the concept of subspecies was introduced by Walter Rothschild [4] in his work “*A revision of the Papilios of the eastern hemisphere, exclusive of Africa*”.

The definition proposed by Rothschild was as follows: “A species with a wider range develops mostly in the different districts into more or less well-characterised local or geographical forms (races), which are termed in this paper subspecies”. Over the years, other subspecies concepts were proposed, e.g., by Amadon [27] and O’Brien and Mayr [28].

The recent concept of subspecies in butterflies was proposed by Braby and co-authors [29], and is as follows: “Subspecies comprise evolving populations that represent partially isolated lineages of a species that are allopatric, phenotypically distinct, have at least one fixed diagnosable character state, and that these character differences are, or assumed to be, correlated with evolutionary independence according to population genetic structure.” In the present research, we follow this concept.

### 2.2. Specimen Sampling

**Figure 1:**
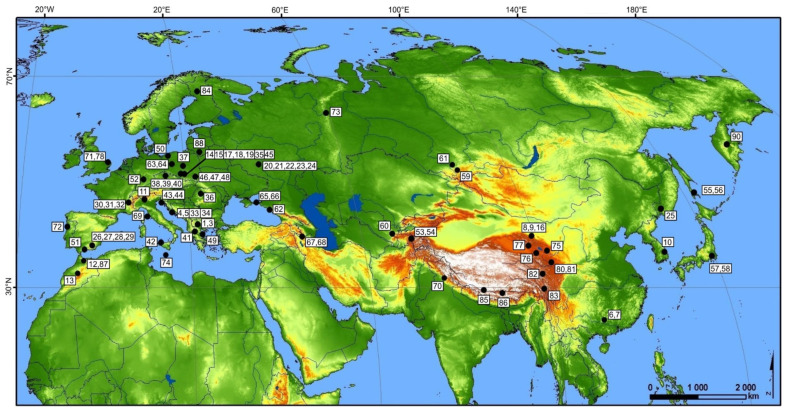
Collecting locality of specimens used in this study.

In this study, 87 specimens sampled in 59 localities that cover the geographic distribution of *P. machaon* in the Palaearctic region were used. Most of the analysed specimens were freshly collected. However, as shown for other insects [30,31,32], mtDNA sequences can be successfully recovered even from old, dried museum specimens. Therefore, such specimens were also used in our analyses. Collecting localities for all specimens (dried and freshly sampled) are listed in Table S2 and shown in Figure 1. 

### 2.3. DNA Extraction and Amplification

The total genomic DNA was extracted from thorax muscle tissues (fresh specimens) or legs (dry museum specimens) using a Sherlock AX kit (A&A Biotechnology, Gdańsk, Poland) following the manufacturer’s protocol. PCR was carried out in a total volume of 50 μL using a ready-to-use mix of PCR Mix Plus (A&A Biotechnology, Gdańsk, Poland) and primer pairs. The primer pairs used in this study were as follows: 16S: LR-J-12887 5′-CCGGTTTGAGCTCAGATC-3′, LR-N-13398 5′-CGCCTGTTTATCAAAAACAT-3′ [33] and REVCB2H 5′-TGAGGACAAATATCATTTTGAGG-3′, REVCBJ 5′-ACTGGTCGAGCTCCAAT-TCATGT-3′ [34].

The PCR for the cytochrome b marker consisted of initial denaturation for 3 min at 94 °C followed by 35 cycles of 30 s at 94 °C, 30 s at 48 °C, 45 s at 72 °C, and the final elongation for 5 min at 72 °C. For 16S rDNA, the protocol was as follows: initial denaturation for 2 min at 95 °C, followed by 37 cycles of 30 s at 95 °C, 30 s at 47 °C, 30 s at 72 °C, and the final elongation for 1 min at 72 °C. The purification of amplicons and sequencing were carried out at A&A Biotechnology, Gdynia, Poland.

All sequences were obtained in the forward and reverse direction, and their correctness was confirmed using the BLAST tool (https://blast.ncbi.nlm.nih.gov/) (accessed on 1 March 2022). The quality of the analysed nucleotide sequences was verified with the Trace Data File Viewer; sequences were aligned using ClustalW (keeping the default parameters) in the MEGA X software [35]. All sequences were deposited in GenBank under accession numbers: ON512348-ON512434 for 16S rDNA and ON529119-ON529205 for the cytochrome b gene.

### 2.4. Molecular Analysis

The nucleotide sequences of cytochrome b were translated to amino acid sequences to check frameshifts and stop codons. Standard genetic diversity, i.e., the number of haplotypes (H), haplotype diversity (hd), nucleotide diversity (ND), the total number of nucleotide differences (TM) and the average number of nucleotide differences (k) were calculated using DnaSp v.6 (Universitat de Barcelona, Spain) [36]. The haplotype networks for each marker were generated using a median-joining network method [37] in PopART v.1.7 software (Allan Wilson Centre Imaging Evolution Initiative, Otago, New Zealand) [38]. The Tajima’s D [39] test was performed using DnaSP v.6 (Universitat de Barcelona, Spain) [36].

The genetic distance between individuals was calculated using a p-distance model in MEGA X software [35]. 

The maximum-likelihood tree combined for cytochrome b and 16S rDNA data was generated using IQ-TREE [40] on the web server [41] with 10,000 replications by the ultrafast bootstrap method [42]. Each gene’s sequences of *Parnassius apollo* L. (KF746065.1) and *Papilio xuthus* L. (EF621724.1) were obtained from GenBank and indicated as the outgroup.

## 3. Results

After purification and sequencing, 87 sequences of cytochrome b (345 bp) and 16S rDNA (468 bp) were obtained. 

Altogether, 22 haplotypes of 16S rDNA were recognised, of which 13 were unique for individual specimens. The most common haplotype (Hap 1) of 16S rDNA was present in 57 specimens and was geographically widely distributed. Overall, the 16S rDNA haplotype diversity for all analysed specimens was 0.5704, and the detected nucleotide diversity was 0.00424. All obtained genetic indices and average pairwise genetic distances for 16S rDNA are presented in Table 1. For the cytochrome b gene, 33 haplotypes were detected. The four most common haplotypes (Hap 2, Hap 3, Hap 4 and Hap 5) were present in 50 specimens, and the unique haplotypes were found in 24 specimens. The obtained nucleotide diversity for cytochrome b was 0.02691, and the overall haplotype diversity was 0.9137. All obtained genetic indices and average pairwise genetic distances for cytochrome b are presented in Table 2.

The list of 16S rDNA and cytochrome b haplotypes and their relationships among analysed specimens and subspecies is presented in Table S2.

**Figure 2:**
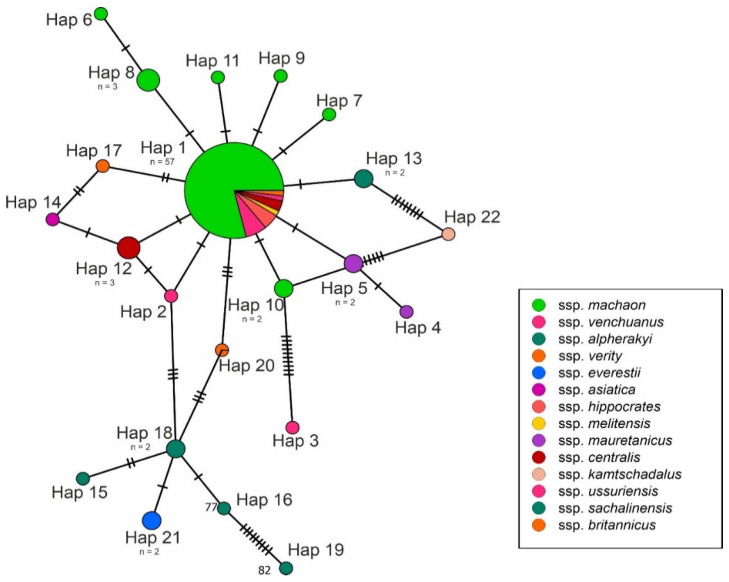
Median-joining haplotype network for 16S rDNA sequences. Different colours are related to subspecies, recognized according to Sturm [3]. The number of individuals with the same haplotype is provided below the haplotype name. Hatch marks along edges represent the number of mutations between nodes. (Hap 17 for 16S rDNA and Hap 25 for cytochrome b), differing from the most common haplotypes only by one and two mutations. The phylogenetic analysis also indicated the lack of sequences distinctiveness of the British specimens of *P. machaon*.

**Figure 3:**
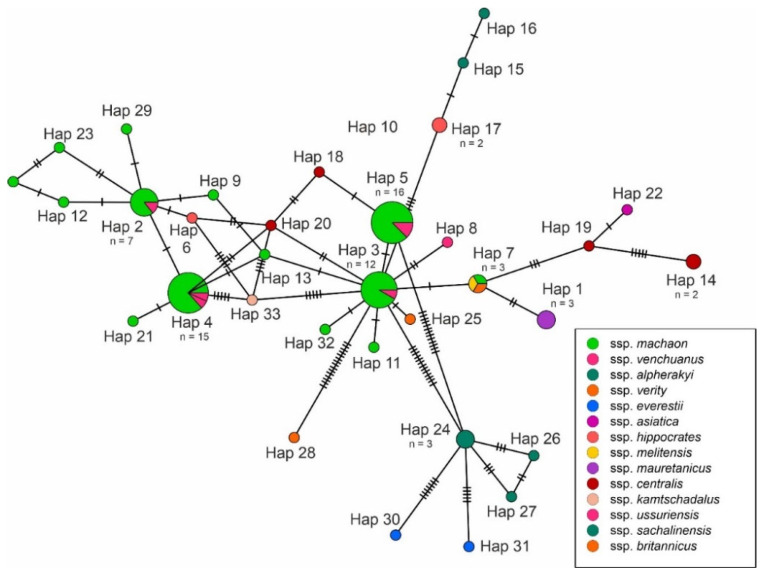
Median-joining haplotype network for cytochrome b sequences. Different colours are related to subspecies, recognized according to Sturm [3]. The number of individuals with the same haplotype is provided below the haplotype name. Hatch marks along edges represent the number of mutations between nodes.

The obtained haplotype networks are presented in Figure 2 and Figure 3. The median-joining haplotype network for 16S rDNA showed a star-like pattern, where the most common haplotype (Hap 1) was located in the centre and surrounded by derived haplotypes. The cytochrome b network showed a similar star-like pattern; however, there was no single central haplotype, but the four most common haplotypes (Hap 2, Hap 3, Hap 4 and Hap 5) were surrounded by derived haplotypes.

The Tajima’s D test for all analysed specimens had a negative value (−2.40567 in 16S rDNA, and −1.70978 for the cytochrome b gene).

**Figure 4:**
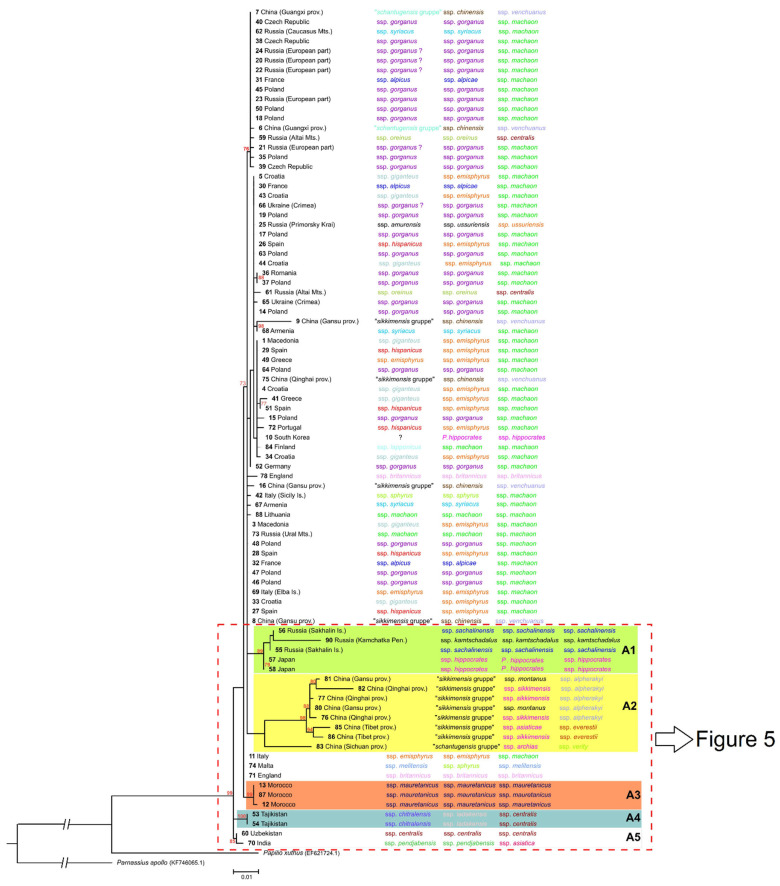
The maximum-likelihood tree derived from a combination of 16S rDNA and cytochrome b sequences. Bootstrap values over 70% are shown next to the branches. The scale bar shows the number of substitutions per site. The individuals on the tree are related to subspecies according to approach of Eller [1] (first column), Seyer [2,7,8,9] (second column) and Sturm [3] (third column).

The maximum-likelihood tree showed a shallow topology (Figure 4). In many cases, specimens representing different subspecies with various geographical origins were located on the same branches. However, a few distinct and highly supported clades were also identified on the ML tree.

**Figure 5:**
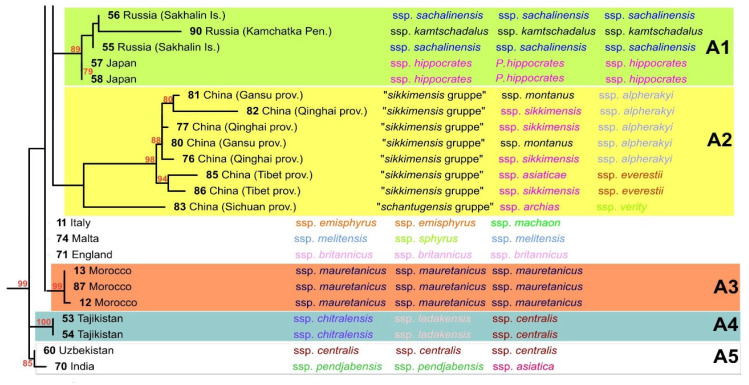
The part of the maximum-likelihood tree (Figure 4) derived from a combination of 16S rDNA and cytochrome b sequences. Bootstrap values over 70% are shown next to the branches. The scale bar showed the number of substitutions per site. The individuals on the tree are related to subspecies according to approach of Eller [1] (first column), Seyer [2,7,8,9] (second column) and Sturm [3] (third column).

Clade A1 contains specimens from Sakhalin (specimens 55, 56), Kamchatka (specimen 90) and Japan (specimens 57, 58). The second clade, A2, contains only specimens from China (from the mountainous regions of Gansu, Qinghai and Tibet). In clade A3, only the specimens from Morocco (specimens 12–13, 87) are included. Two smaller clades, A4 and A5, contain two specimens from Tajikistan (specimens 53, 54), and the specimen from Uzbekistan (specimen 60) and India (specimen 70), respectively (see the Figure 5).

## 4. Discussion

The Old World swallowtail is known for its tendency to change the wing colour pattern. Due to the great interest of collectors and amateur entomologists, this kind of morphological variability led to descriptions of over a hundred colour forms, aberrations and subspecies. This situation is not unprecedented because a similar problem with the extensive and ambiguous intraspecific classification occurs in other Papilionidae species [43,44,45] and common butterfly species, e.g., *Aglais urticae* L. [46].

Butterflies, especially representatives of the family Papilionidae, are among insects’ most popular collecting specimens [47,48]. The researchers’ interest was usually focused on significant, colourful specimens, to which, undoubtedly, the Old World swallowtail belonged. 

The popularity of *P. machaon* specimens collected in the 19th century, the lack of rules and standardised procedures for describing and naming new taxa, and the lack of scientific communication at that time caused this species nomenclature to be very complicated. Various morphological groups of individuals have been defined as forms, aberrations and varieties, which were uncritically raised to subspecies even in almost all subsequent publications [1,2,5].

Moreover, in the late nineteenth and early twentieth centuries, attempts to crossbreed different species were very common, as well as experiments on the impact of temperature and other indices on the colour pattern of butterfly wings [49]. The 19th century was also a time of commonly operating entomological traders [50,51]. The owners of entomological companies, directing their offers to collectors, made the price lists more attractive for marketing and commercial purposes, artificially creating irrelevant names from a taxonomic point-of-view. Those names were later introduced directly from the catalogues into scientific papers and were repeated in subsequent publications [6]. In addition, the same Latin names of aberrations, forms and subspecies of *P. machaon* are sometimes attributed to different authors [1,2,3]. There are also cases of unjustified changes in the original Latin name spelling [1,2,52]. When all the above facts are considered, it is challenging to provide the exact number of described aberrations, forms and subspecies of *P. machaon*.

Depending on the author, the number of the Old Word swallowtail subspecies occurring in Europe and North Africa ranges from four [3,53] to twelve [2,7,11], or in the oldest study by Eller [1], even thirteen. However, Tshikolovets [54] did not recognise any subspecies of *P. machaon* in Europe, considering the morphological variability of specimens constituting European populations too low for such taxonomic actions.

Below, we discuss the results of our studies of specimens of *P. machaon* concerning the geographical region where they occur.

### 4.1. The British Isles

Regardless of the number of distinguished subspecies, most researchers agreed that the population of the Old World swallowtail from the British Isles represents the endemic subspecies ssp. *britannicus* [1,2,3,53]. The original diagnosis of this subspecies given by Seitz [6] was very laconic and limited to a few words: “ein breit und tief Schwarz gezeichneter machaon mit besonders breiter, samtschwarzer Submarginalbinde” and lithography. The cited description shows that the only considered characteristics were a darker colour of the veins (deep black) and a wide submarginal band.

Our results on individuals from the British Isles showed that one specimen (no 71) represented identical haplotypes (Hap 1, Hap 17 for 16S rDNA and cytochrome b, respectively), as individuals from other regions of continental Europe. They were part of a large monophylum, including many other continental European individuals (e.g., from Croatia, Italy, France or Spain). The second British specimen represented unique haplotypes.

The analysis did not show the distinctiveness of the British subspecies from the other European subspecies as proposed by many authors [1,2,3,7,53,54,55]. The haplotype analyses and the location of European specimens on phylogenetic trees indicate that despite the significant geographical distances between populations, there is the possibility of a flow of genetic information throughout Europe (including the British Isles). Thus, our results suggest that the Old World swallowtail is an excellent flyer, and its individuals have no problem moving large distances, enabling the exchange of genes within one large genetic pool throughout Europe and Eurasia. Indirect evidence for this may be that the migration rate of *P. machaon* in the case of North American populations was estimated at 4.3 km per day in upwind conditions (after Sperling and Harrison [19]). In favourable weather conditions (downwind), this rate is probably higher. Combined with the long flight period of imagines and the occurrence of up to three generations a year [55], it may be possible to transfer genetic information over considerable distances. On the other hand, it is hypothetically possible to transfer preimaginal stages with the food plants, as was in the case of other butterflies [56,57,58].

### 4.2. North Africa

The Old World swallowtail populations from North Africa are primarily classified into the subspecies ssp. *mauretanicus* [1,2,3]. However, Seyer [2] distinguished the additional subspecies ssp. *maxima* in that region of Africa. This is regarded as a synonym of the subspecies ssp. *mauretanicus* by Eller [1] and Sturm [3].

Moreover, *P. saharae* Oberthür, 1879, a species related to *P. machaon* was also reported from North Africa [59,60]. It was described as a subspecies of *P. machaon* [59], and this taxonomic rank was kept for it by some authors [1,2]. However, Pittaway et al. [60] raised it to the species rank and this was also used by some other lepidopterologists [3,49,60,61]. The morphological features distinguishing *P. machaon* from *P. saharae* concern different antennal segments *(P. saharae* has 30–31 segments, while *P. machaon* has 33–36 segments [55,60]). The distribution area of *P. saharae* is not limited only to North Africa, as Moonen [61] and Sturm [3] also reported this species from Sicily.

All dendrograms have identified the Moroccan individuals as constituting one clade (A3). The haplotype analyses also confirmed their distinctiveness. The Moroccan specimens held two unique haplotypes (Hap 4 and Hap 5) for the 16S rDNA sequences and a unique haplotype (Hap 1) for the cytochrome b gene. Similar results for *Iphiclides podalirius* L. (the other Papilionidae species), which inhabits both sides of the Gibraltar strait, were obtained by Wiemers and Gottsberger [62] and Dincă et al. [63], based on COI sequence analysis. They showed no significant differences in the sequence of this gene between *I. podarilius* specimens inhabiting various regions of Europe. Still, they proved the existence of apparent differences between the Moroccan and Spanish populations. They indicated that even a 14-kilometre strait separating Africa and Europe might constitute a barrier to mixing the pool gene of these two populations [62].

### 4.3. Asia (Japan, Russia)

The Japanese populations of *P. machaon* are usually considered as the subspecies *hippocrates* [1,3,23], but Seyer [8,9,14] regarded those populations as a separate species, *P. hippocrates*. According to Sturm [3] the range of the subspecies *hippocrates* includes Japan, Korea and the Kuril Islands. However, Seyer [13,14] upraised it to a full species, and placed two other subspecies within its synonymy (i.e., ssp. *ussurensis* from the Primorsky Krai in Russia and ssp. *sachalinensis* from Sakhalin), at the same time extending the area of its occurrence to these two regions. 

The haplotype analyses showed that the specimen from Primorsky Krai in Russia, representing the subspecies *ussurensis*, did not differ from individuals sampled throughout the entire Eurasian range of *P. machaon*, and held the most common haplotypes both for 16S rDNA (Hap 1) and cytochrome b (Hap 4). The results obtained from haplotype analysis of specimens sampled in Korea and Japan (ssp. *hippocrates*) did not confirm their distinctiveness in 16S rDNA sequences (they represented the same haplotype—Hap 1) but confirmed differences in the cytochrome b gene, where two different haplotypes were detected (Hap 6, Hap 17).

The specimens from Sakhalin Island represented the subspecies *sachalinensis* and the haplotype analysis indicated its distinctiveness. Unique haplotypes were obtained in both cases (16S rDNA and cytochrome b). Moreover, they formed a single well-defined clade on the phylogenetic trees resulting from analyses of both 16S rDNA and cytochrome b sequences.

Our results are congruent with the studies of Miyakawa et al. [23,24], which analysed Japanese and East Asian populations of *P. machaon* based on the sequences of the ND5 mitochondrial gene. They confirmed the differences between the insular Japanese and mainland Asian populations and indicated the close relationship between the Sakhalin and Japanese specimens [23,24].

According to the intraspecific classification accepted by Eller [1,64], Seyer [9], Tuzov [65] and Sturm [3], specimens from the Kamchatka Peninsula should be regarded as representing the ssp. *kamtschadalus*. Depending on the author, the specimens from the Altai Mountains belong to three different subspecies, i.e., ssp. *oreinus* [1,9], ssp. *orientis* Tuzov [56] and ssp. *centralis* [3]. Contrary to all other authors, Tshikolovets et al. [66] regarded individuals from Altai as the nominative subspecies *P. machaon machaon*.

Our analysis of the 16S rDNA gene indicated no genetic differences between the Altai individuals and the specimens from the other parts of Russia and the whole of Europe. However, the analysis of cytochrome b haplotypes generated two unique haplotypes (Hap 18 and Hap 20), which differed from most common haplotypes (Hap 3, Hap 4, and Hap 5) by one and two mutations.

When the Kamchatka specimen is considered, its placement on the phylogenetic trees and the haplotype analysis indicates the phylogenetic distinctiveness from other specimens. The specimen from Kamchatka formed a clade (A1) with the individuals from Japan and Sakhalin. Our results differ from those of Miyakawa et al. [23], where specimens widespread across the Palearctic constituted a single clade. However, the specimens from Japan and Sakhalin were placed outside this monophyletic group in our study.

### 4.4. Asia (China)

Chinese populations of *P. machaon* were the subject of several studies [1,9,14,67,68,69,70], where the status of many subspecies was changed because of synonymisation or raising to the species rank. Therefore, the number of Chinese subspecies of *P. machaon* varied depending on the author.

Lee [68] recognised only three species of the *P. machaon* group in China, namely *P. machaon*, *P. annae* and *P. verity*, without any subspecies. Moreover, he synonymised *P. machaon sikkimensis* with *P. annae*, and *P. machaon birmanicus* with *P. verity*. Based on this concept [68], the specimens from Guangxi province (6 and 7), Gansu (8, 9 and 16) and Qinghai (75), which we analysed, represent *P. machaon*. On the other hand, the specimens from the Qinghai province (76, 77, 82) and Gansu (80, 81) and individuals from Tibet (85, 86) belong to *P. annae*. In contrast, the specimen from Sichuan (83) should be assigned to *P. verity*.

Our haplotype analyses of the 16S rDNA and cytochrome b sequences in Chinese specimens showed that some are characterised by haplotypes typical of individuals of the entire palaearctic range of *P. machaon*. However, some (e.g., specimens from the mountainous regions of Gansu, Qinghai and Tibet) are often entirely different from other haplotypes. The genetic differences between the specimens from the mountain regions of Gansu, Qinghai and Tibet (76, 77, 80, 81, 82, 83, 85, 86), and the other specimens representing the entire Palearctic range of *P. machaon* are distinctly marked. These results are supported by the ML phylogram, where the specimens from mountain regions of Gansu, Qinghai and Tibet form a separate clade. Nevertheless, a group of five specimens (7, 8, 9, 16 and 75) does not show significant genetic variation, and the genetic distances of these specimens to the European ones are low.

Results of all our analyses of the Chinese specimens coincide with the concepts of *P. machaon* intraspecific division proposed by Lee [68] and Huang [70], where Chinese individuals were classified into three groups (*machaon* group, *sikkimensis* group and *verity* group). Most importantly, we have confirmed the existence of three separate populations in China, among which the exchange of genes is limited. Presumably, an isolation mechanism exists similar to that mentioned by Gaonkar [69] concerning another subspecies *P. machaon*, where populations above 4000 m a.s.l. are isolated from lowland populations by a layer of monsoon clouds. Clouds that are more than one kilometre thick prevent the vertical movement of these butterflies. Moreover, the flight period of imagines of high-mountain populations is short, impacting the possible exchange of genetic information.

### 4.5. Asia (India, Tajikistan and Uzbekistan)

It should also be considered that individuals from northern parts of India, Tajikistan and Uzbekistan are geographically the most related specimens to the Chinese ones. According to the literature data, the specimens from populations in these countries can be considered as several different subspecies. In the case of *P. machaon* from Tajikistan and Uzbekistan, it is impossible to deliver a consistent intraspecific classification. 

According to Eller [1], our specimens from Tajikistan can be classified as ssp. *chitralensis*. In Seyer’s [8] approach, those specimens are representatives of the subspecies ssp. *ladakensis* (a form of *chitralensis*), and according to Tuzov [65] and Sturm [3], Tajik specimens represent the subspecies ssp. *centralis*. This subspecies should include a specimen from Uzbekistan from the vicinity of Samarkand (*locus typicus* for the var. *centralis* described by Staudinger [71]. 

The specimen from India was classified as ssp. *pendjabensis* by Eller [1] and Seyer [9], but as ssp. *asiatica* by Sturm [3]. Results of our study indicate the specimen from India is distinct from Chinese specimens, holding the unique haplotypes for 16S rDNA (Hap 14) and cytochrome b (Hap 22). Its position on the phylogenetic tree suggests a relationship to the specimen from Uzbekistan (60). The cytochrome b haplotype network analysis showed that the Indian specimen sequence differed from that of the specimen from Uzbekistan by only a single mutation. A similar result concerning the haplotype network of 16S rDNA was analysed. The sequence of the Indian specimen differed from the specimens from Tajikistan and Uzbekistan (which have the same haplotype Hap 12) also by only one mutation. However, the Tajik specimens formed one clade (A4) on the resulting ML phylogenetic tree.

## 5. Conclusions

Our analysis showed that various authors’ previously proposed intraspecific classifications (see Tables S1 and S2) do not correlate with variability in the mitochondrial DNA sequences. Kato and Yagi [72] and Todisco [43] observed a similar lack of dependence for other Papilionidae. 

High haplotype diversity combined with low nucleotide diversity can suggest rapid demographic expansion from a small population [73] or multiple refuges and contact of haplotypes [74].

The Tajima’s D statistic was negative in our results for 16S rDNA and cytochrome b markers. The negative Tajima’s D statistics also suggest an excess of low-frequency mutations, for example, after a recent population expansion [75,76]. On the other hand, the most common haplotypes are distributed within the entire palaearctic range of *P. machaon*, suggesting high gene flow [46].

According to our results, all *P. machaon* subspecies recognised so far from the European territories show no significant genetic distances and should probably not be treated as separate infraspecific taxa. On the other hand, ssp. *mauretanicus* (northern Africa), ssp. *kamtschadalus* (Kamchatka) and ssp. *hippocrates* (Japan) are distinctly divergent genetically, suggesting their status as separate subspecies.

However, we acknowledge the limitations of our results which reflect only the maternal gene inheritance and we are aware that our results should be supported and tested with additional nuclear markers in future.

Moreover, we suggest a necessity for further molecular studies on *P. machaon* distributed in China and adjacent territories, including as many specimens as possible from different regions.

## Figures and Tables

**Table 1:** Standard genetic indices and average pairwise genetic distance of 16S rDNA marker.

Subspecies *	n	H	uH	hd	S	V	ND	TM	*k*	Dist
All	87	22	-	0.5704	39	39	0.00424	41	1.982	-
ssp. *machaon*	54	7	6	0.3054	6	6	0.00077	6	0.361	0.00077
ssp. *alpherakyi*	5	4	3	0.9000	11	11	0.01026	11	4.800	0.01026
ssp. *asiatica*	1	1	1	-	-	-	-	-	-	-
ssp. *britanicus*	2	2	1	1.0000	2	2	0.00427	2	2.000	0.00427
ssp. *centralis*	5	2	1	0.6000	1	1	0.00128	1	0,600	0.00128
ssp. *everestii*	2	1	2	0.0000	0	0	0.00000	0	0.000	0
ssp. *hippocrates*	3	1	0	0.0000	0	0	0.00000	0	0.000	0
ssp. *kamtschadalus*	1	1	1	-	-	-	-	-	-	-
ssp. *mauretanicus*	3	2	2	0.6670	1	1	0.00142	1	0.667	0.00142
ssp. *melitensis*	1	1	0	-	-	-	-	-	-	-
ssp. *sachalinensis*	2	1	1	0.0000	0	0	0.00000	0	0.000	0
ssp. *ussuriensis*	1	1	0	-	-	-	-	-	-	-
ssp. *venchuanus*	6	3	2	0.6000	12	12	0.00855	12	4.000	0.00855
ssp. *verity*	1	1	1	-	-	-	-	-	-	-

n—number of analysed sequences; H—number of haplotypes; uH—number of unique haplotypes for each subspecies; *hd*—haplotype diversity, S—number of polymorphic (segregating) sites; V—number of variable sites; ND—nucleotide diversity; TM—total number of mutations; *k*—the average number of nucleotide differences; Dist—average pairwise genetic distance within subspecies; *—subspecies according to Sturm [3].

**Table 2:** Standard genetic indices and average pairwise genetic distance of 16S rDNA marker.

Subspecies *	n	H	uH	hd	S	V	ND	TM	*k*	Dist
All	87	33	-	0.9137	55	55	0.02691	59	9.229	-
ssp. *machaon*	54	14	9	0.833	10	10	0.00606	10	2.079	0.00606
ssp. *alpherakyi*	5	3	2	0.700	4	4	0.00583	4	2.000	0.00583
ssp. *asiatica*	1	1	1	-	-	-	-	-	-	-
ssp. *britanicus*	2	2	1	1.000	2	2	0.00583	2	2.000	0.00583
ssp. *centralis*	5	4	4	0.900	9	9	0.01516	9	5.200	0.01516
ssp. *everestii*	2	2	2	1.000	8	8	0.02332	8	8.000	0.02330
ssp. *hippocrates*	3	2	2	0.667	7	7	0.01361	7	4.667	0.01360
ssp. *kamtschadalus*	1	1	1	-	-	-	-	-	-	-
ssp. *mauretanicus*	3	1	1	0.000	0	0	0	0	0	0.00000
ssp. *melitensis*	1	1	0	-	-	-	-	-	-	-
ssp. *sachalinensis*	2	2	2	1.000	1	1	0.00292	1	1.000	0.00292
ssp. *ussuriensis*	1	1	0	-	-	-	-	-	-	-
ssp. *venchuanus*	6	6	1	0.933	6	6	0.00758	6	2.600	0.00758
ssp. *verity*	1	1	1	-	-	-	-	-	-	-

n—number of analysed sequences; H—number of haplotypes; uH—number of unique haplotypes for each subspecies; *hd*—haplotype diversity, S—number of polymorphic (segregating) sites; V—number of variable sites; ND—nucleotide diversity; TM—total number of mutations; *k*—the average number of nucleotide differences; Dist—average pairwise genetic distance within subspecies; *—subspecies according to Sturm [3].

## Data Availability

Sequences used in this study were deposited in NCBI GenBank under accession numbers: ON512348-ON512434 and ON529119-ON529205.

## Supplementary Materials

The following supporting information can be downloaded at: https://www.mdpi.com/article/10.3390/insects13080752/s1, Table S1. List of colour forms, aberrations and subspecies of *Papilio machaon* L.; Table S2. The summary of specimens used in this study and their relationships to different subspecies and haplotypes.
