# Supplementary material for: One Species, Hundreds of Subspecies? New Insight into the Intraspecific Classification of the Old World Swallowtail (Papilio machaon Linnaeus, 1758) Based on Two Mitochondrial DNA Markers
Source: Insects. 2022 Aug 21;13(8):752. doi: 10.3390/insects13080752 (PMC9409990; doi:10.3390/insects13080752)
Supplement: Supplementary file 1 [file insects-13-00752-s001.zip › Table S2.pdf]

Table S2. The summary of specimens used in this study, and their relations to different subspecies and haplotypes.

Country	Locality	Specimen no. and type	Year	*n*	Subspecies			Haplotype symbol	
					Eller (1936)	Seyer*	Sturm (2017)	16S rDNA	Cytb
Armenia	Zangezur range, Vahravar vill.	67 (D), 68 (D)	2010	2	ssp. *syriacus*	ssp. *syriacus*	ssp. *machaon*	Hap 10	Hap 3<br>Hap 4
China	Gansu prov., Zhangye, 2600 mts.	8 (D), 9 (D),<br>16 (D)	2014	3	"*sikkimensis* gruppe"	ssp. *chinensis*	ssp. *venchuanus*	Hap 1<br>Hap 3	Hap 3<br>Hap 4<br>Hap 8
	Gansu prov., Xiahe country, 2500 mts.	80 (D), 81 (D)	2018	2	"*sikkimensis* gruppe"	ssp. *montanus*	ssp. *alpherakyi*	Hap 18	Hap 24<br>Hap 26
	Guangxi prov., Lingui District, Guilin city,	6 (D), 7 (D)	2014	2	"*schantugensis* gruppe"	ssp. *chinensis*	ssp. *venchuanus*	Hap 1<br>Hap 2	Hap 5
	Sichuan prov., Batang, 2600 mts.	83 (D)	2018	1	"*schantugensis* gruppe"	ssp. *archias*	ssp. *verity*	Hap 20	Hap 28
	Tibet prov., Xigaze area, N. Kangmar, 5000 mts.	86 (D)	2018	1	"*sikkimensis* gruppe"	ssp. *sikkimensis*	ssp. *everestii*	Hap 21	Hap 31
	Tibet prov., Xigaze area, S from Saga city, 5000 mts.	85 (D)	2018	1	"*sikkimensis* gruppe"	ssp. *asiaticae*	ssp. *everestii*	Hap 21	Hap 30
	Qinghai prov, Dulan county, Xiariha town, 4000 mts.	77 (D)	2018	1	"*sikkimensis* gruppe"	ssp. *sikkimensis*	ssp. *alpherakyi*	Hap 16	Hap 24
	Qinghai prov., Guide, Qunfia Pass, 3500 m	76 (D)	2018	1	"*sikkimensis* gruppe"	ssp. *sikkimensis*	ssp. *alpherakyi*	Hap 15	Hap 24
	Qinghai prov., Hualong country, 20 km E of Zhaba town,<br>2500-3000 mts.	75 (D)	2017	1	"*sikkimensis* gruppe"	ssp. *chinensis*	ssp. *venchuanus*	Hap 1	Hap 2
	Qinghai prov., Yushu area, Zadoi county, 4000 mts.	82 (D)	2017	1	"*sikkimensis* gruppe"	ssp. *sikkimensis*	ssp. *alpherakyi*	Hap 19	Hap 27
Croatia	Istria, Novigrad	43 (D), 44 (D)	2000	2	ssp. *giganteus*	ssp. *emisphyrus*	ssp. *machaon*	Hap 1	Hap 4
	Hvar Is. Hvar City	34 (D)	1998	1	ssp. *giganteus*	ssp. *emisphyrus*	ssp. *machaon*	Hap 1	Hap 9
	Omis	33 (D)	1999	1	ssp. *giganteus*	ssp. *emisphyrus*	ssp. *machaon*	Hap 1	Hap 3
	Tucepi, Makarska	4 (D), 5 (D)	2001	2	ssp. *giganteus*	ssp. *emisphyrus*	ssp. *machaon*	Hap 1	Hap 2<br>Hap 4
Czech Rep.	Pardubice	38 (F), 39 (F),<br>40 (F)	2014	3	ssp. *gorganus*	ssp. *gorganus*	ssp. *machaon*	Hap 1<br>Hap 9	Hap 5
Finland	Rovaniemi Reg., Near Tapionkyla	84 (D)	2014	1	ssp. *lapponicus*	ssp. *machaon*	ssp. *machaon*	Hap 1	Hap 29
France	Vif (Isère), Auvergne-Rhône-Alpes	30 (F), 31 (F),<br>32 (F)	2015	3	ssp. *alpicus*	ssp. *alpicae*	ssp. *machaon*	Hap 1	Hap 3<br>Hap 4<br>Hap 5
Germany	Fiderstadt, Stuttgart	52 (D)	2011	1	ssp. *gorganus*	ssp. *gorganus*	ssp. *machaon*	Hap 1	Hap 13
Greece	Despotis	41 (D)	2012	1	ssp. *giganteus*	ssp. *emisphyrus*	ssp. *machaon*	Hap 11	Hap 10
	Karitsa	49 (F)	2015	1	ssp. *emisphyrus*	ssp. *emisphyrus*	ssp. *machaon*	Hap 1	Hap 2
India	Himachal Pradesh, Kullu Tehsil, Pulga, 2400 mts.	70 (D)	1999	1	ssp. *pendjabensis*	ssp. *pendjabensis*	ssp. *asiatica*	Hap 14	Hap 22
Italy	Elba Is., Lacona – Monte Grosso	69 (D)	2013	1	ssp. *emisphyrus*	ssp. *emisphyrus*	ssp. *machaon*	Hap 1	Hap 3
	Provincia di Lecco, Regione Lombardia, Civate	11 (F)	2014	1	ssp. *emisphyrus*	ssp. *emisphyrus*	ssp. *machaon*	Hap 1	Hap 7
	Sicilia Is. Prov. Palermo, Bosco Della Fieuzza, Fieuzza env.	42 (D)	2016	1	ssp. *sphyrus*	ssp. *sphyrus*	ssp. *machaon*	Hap 1	Hap 11
Japan	Kanagawa pref., Yokohama city	57 (D), 58 (D)	2016	2	ssp. *hippocrates*	*P. hippocrates*	ssp. *hippocrates*	Hap 1	Hap 17
Lithuania	Varena reg. Kasetos vill.	88 (F)	2018	1	ssp. *machaon*	ssp. *machaon*	ssp. *machaon*	Hap 1	Hap 32
Macedonia	Gevgelia env.	1 (D), 3 (D)	2014	2	ssp. *giganteus*	ssp.*emisphyrus*	ssp. *machaon*	Hap 1	Hap 2<br>Hap 3
Malta	Rabbat	74 (D)	1999	1	ssp. *melitensis*	ssp. *sphyrus*	ssp. *melitensis*	Hap 1	Hap 7

Morocco	Rif occidental, Forêt de Chouihat, S djebel Tisouka, N Chefchaouen	12 (D), 87 (D)	2014	2	ssp. *mauretanicus*	ssp. *mauretanicus*	ssp. *mauretanicus*	Hap 4<br>Hap 5	Hap 1
	Haut Atlas central, Imi-n-Ifri, Demnate, assif Tissilt	13 (D)	2014	1	ssp. *mauretanicus*	ssp. *mauretanicus*	ssp. *mauretanicus*	Hap 5	Hap 1
Poland	Kraków-Częstochowa Upland: Olsztyn	17 (F), 18(F), 19 (F)	2014	3	ssp. *gorganus*	ssp. *gorganus*	ssp. *machaon*	Hap 1	Hap 4<br>Hap 5
	Małopolska Upland: Łódź	37 (F)	2015	1	ssp. *gorganus*	ssp. *gorganus*	ssp. *machaon*	Hap 8	Hap 4
	Pomeranian Lakeland: Połczyn-Zdrój	50 (F)	2015	1	ssp. *gorganus*	ssp. *gorganus*	ssp. *machaon*	Hap 1	Hap 5
	Sandomierska Lowland: Leżajsk	46 (F), 47 (F), 48 (F)	2016	3	ssp. *gorganus*	ssp. *gorganus*	ssp. *machaon*	Hap 1	Hap 3
	Upper Silesia: Imielin	35 (F)	2015	1	ssp. *gorganus*	ssp. *gorganus*	ssp. *machaon*	Hap 8	Hap 5
	Upper Silesia: Lubliniec	14 (F)	2014	1	ssp. *gorganus*	ssp. *gorganus*	ssp. *machaon*	Hap 1	Hap 4
	Upper Silesia: Olesno	45 (F)	2016	1	ssp. *gorganus*	ssp. *gorganus*	ssp. *machaon*	Hap 1	Hap 5
	Upper Silenia: Szklarnia near Lubliniec	15 (F)	2014	1	ssp. *gorganus*	ssp. *gorganus*	ssp. *machaon*	Hap 6	Hap 2
	Wielkopolsko-Kujawska Lowland: Czempin	64 (F)	2017	1	ssp. *gorganus*	ssp. *gorganus*	ssp. *machaon*	Hap 1	Hap 2
	Wielkopolsko-Kujawska Lowland: Poznań	63 (F)	2017	1	ssp. *gorganus*	ssp. *gorganus*	ssp. *machaon*	Hap 1	Hap 4
Romania	Clui Napoca	36 (F)	2014	1	ssp. *gorganus*	ssp. *vargaianus*	ssp. *machaon*	Hap 8	Hap 4
Russia	Altai, Katun riv., Chemal vill., 430 mts.	61 (D)	2016	1	ssp. *oreinus*	ssp. *oreinus*	ssp. *centralis*	Hap 1	Hap 20
	Altai mts., Ongudai distr., Cheki-Taman pass., 1250 mts.	59 (D)	2015	1	ssp. *oreinus*	ssp. *oreinus*	ssp. *centralis*	Hap 1	Hap 18
	Caucasus mts., Sochi reg	62 (D)	2015	1	ssp. *syriacus*	ssp. *syriacus*	ssp. *machaon*	Hap 1	Hap 5
	Kamchatka Peninsula, Esso vill.	90 (D)	2014	1	ssp. *kamtschadalus*	ssp. *kamtschadalus*	ssp. *kamtschadalus*	Hap 22	Hap 33
	North Ural, Kvarkush Mr. Range	73 (D)	2014	1	ssp. *machaon* ?	ssp. *machaon*	ssp. *machaon*	Hap 1	Hap 3
	Primorski kraj, Anuchinsky distr., Novogordeevka vill.	25 (D)	2015	1	ssp. *amurensis*	ssp. *ussuriensis*	ssp. *ussuriensis*	Hap 1	Hap 4
	Sakhalin Is., Cape Kril'on, Malikna vill.,	55 (D), 56 (D)	2012	2	ssp. *sachalinensis*	ssp. *sachalinensis*	ssp. *sachalinensis*	Hap 13	Hap 15<br>Hap 16
	Voronezh distr.	20 (D), 21 (D), 22 (D) ,23 (D), 24 (D)	2014	5	ssp. *gorganus* ?	ssp. *gorganus*	ssp. *machaon*	Hap 1<br>Hap 7	Hap 5
Portugal	Aveiro	72 (D)	2011	1	ssp. *hispanicus*	ssp. *emisphyrus*	ssp. *machaon*	Hap 1	Hap 23
South Korea	Andong, Gyeongsangbuk-Do	10 (D)	2014	1	?	*P. hippocrates*	ssp. *hippocrates*	Hap 1	Hap 6
Spain	Granada, La Cruz	26 (F), 27 (F), 28 (F), 29 (F)	2015	4	ssp. *hispanicus*	ssp. *emisphyrus*	ssp. *machaon*	Hap 1	Hap 2<br>Hap 3<br>Hap 4
	Torremolinos	51 (D)	2015	1	ssp. *hispanicus*	ssp. *emisphyrus*	ssp. *machaon*	Hap 1	Hap 12
Tajikistan	Darvaz mts., Kugireiu range, Kalaishum city evnv., near Afghanistan border	53 (D), 54 (D)	2015	2	ssp. *chitralensis*	ssp. *ladakensis*	ssp. *centralis*	Hap 12	Hap 14
Ukraine	Crimea Peninsula, Kerch Peninsula, Lovovo vill.	65 (D), 66 (D)	2015	2	ssp. *gorganus* ?	ssp. *gorganus*	ssp. *machaon*	Hap 1	Hap 4<br>Hap 21
United Kingdom	Monk's Wood, Combr. Shira	71 (D)	1990	1	ssp. *britannicus*	ssp. *britannicus*	ssp. *britannicus*	Hap 1	Hap 7
	Norfolk, Barton Broad	78 (D)	1981	1	ssp. *britannicus*	ssp. *britannicus*	ssp. *britannicus*	Hap 17	Hap 25
Uzbekistan	Altai mts., Zeravshan ridge, Samarkand distr., Urgut, 2400 mts.	60 (D)	2014	1	ssp. *centralis*	ssp. *centralis*	ssp. *centralis*	Hap 12	Hap 19

F – fresh specimen; D – dry specimen; Seyer*: (see References)

References:

1. Eller, K. Die Rassen von Papilio machaon L.; E.J. Brill: Leiden, **1936**; 96 p.

2. Seyer, H. Versuch einer revision der Papilio machaon-subspezies in der westlichen Paläarktis. Mitteilungen der Entomologischen Gesellschaft Basel **1974**, 24(2), 64-90.

3. Seyer, H. Versuch einer revision der Papilio machaon-subspezies in der ostlichen Paläarktis. Mitteilungen der Entomologischen Gesellschaft Basel **1976**, 26(3), 65-87.

4. Seyer, H. Versuch einer revision der Papilio machaon-subspezies in der ostlichen Paläarktis. Mitteilungen der Entomologischen Gesellschaft Basel **1976**, 26(4), 97-145.

5. Sturm, R. Butterflies of the World, Part 45: Papilionidae XVI: Illustrated Checklist of Papilio machaon - group, Iphiclides po-dalirius, and Papilio alexanor. Goecke & Evers Verlag, **2017**; 45 p.
